# Association of triglyceride-glucose index with the prevalence of cardiovascular disease in malnourished/non-malnourished patients: a large cross-sectional study

**DOI:** 10.3389/fcvm.2023.1306415

**Published:** 2023-11-28

**Authors:** Xiaobo Jiang, Jiabin Tu, Sicong Chen, Yanbin Zhang, Weilong Qiu, Kaihong Chen, Liling Chen, Bo Wu

**Affiliations:** Department of Cardiology, Longyan First Affiliated Hospital of Fujian Medical University, Longyan, China

**Keywords:** triglyceride-glucose index, cardiovascular disease, malnutrition, insulin resistance, NHANES

## Abstract

**Background:**

Numerous investigations have demonstrated a strong association between the TyG (triglyceride-glucose) index, which is derived from lipid and glucose levels in the bloodstream, and the onset and progression of cardiovascular diseases (CVD). Blood glucose and blood lipids are affected by nutritional status, and few studies have explored whether the correlation between TyG index and the risk of CVD is affected by nutritional status.

**Aims:**

To investigate the connection between TyG index and the risk of CVD among individuals with varying nutritional statuses.

**Method:**

A total of 19,847 were included in the analysis, of which 15,955 participants were non-malnourished and 3,892 patients were malnourished. According to the TyG index quartile, the patients were categorized into four groups. Logistic regression analysis and restricted cubic spline was used to study the relationship between TyG index and the risk of CVD in normal and malnourished populations.

**Results:**

The results of the restricted cubic spline showed that the TyG index was positively associated with the risk of CVD in the non-malnourished population. The TyG index showed a U-shaped association with the risk of CVD in malnourished people. The result is consistent with that of logistic regression (Malnutrition: Group 2: OR: 1.14; 95% CI: 0.85–1.53; Group 3: OR: 1.36; 95% CI: 1.03–1.79; Group 4: OR: 1.72; 95% CI:1.31–2.25, *P* for trend <0.001; Non-malnutrition: Group 2: OR: 0.82; 95% CI: 0.46–1.48; Group 3: OR: 0.88; 95% CI: 0.49–1.57; Group 4: OR: 1.45; 95% CI:0.83–2.52, *P* for trend =0.067).

**Conclusions:**

The association between the TyG index and the risk of CVD varied depending on the nutritional states. When using TyG index to assess the risk of CVD, stratification combined with nutritional status helps to more accurately screen patients at high risk of CVD.

## Introduction

The mortality attributed to cardiovascular disease (CVD) is now widely recognized as a major global health concern, accounting for approximately 33% of all documented deaths worldwide (1). “Extensive research has shown that individuals with a history of CVD conditions face a significantly higher risk of experiencing CVD-related fatalities compared to those without such a medical history (2). Consequently, implementing preventive strategies at an early stage has emerged as a crucial approach in mitigating CVD mortality.

Numerous studies have shown that the presence of insulin resistance plays a role in the development of CVD. Individuals with elevated levels of insulin resistance are at a higher risk of experiencing CVD (3, 4). Furthermore, the triglyceride-glucose (TyG) index, a metric for assessing insulin resistance using blood glucose and lipids, has shown its link to the onset and advancement of CVD. As noted by Wang et al., the TyG index proved to be a reliable predictor of CVD in the general population (5). Su et al. put forth the idea that the TyG index has the potential to forecast CVD events in patients diagnosed with type 2 diabetes mellitus (DM) (6).

It is worth noting that the TyG index was originally developed and validated in healthy people (7), and the effect of nutritional status on blood sugar and lipids was not considered at the time of development. Malnutrition is often closely related to hypoglycemia and low blood lipids (8–10). Therefore, whether the TyG index calculated by blood glucose and blood lipid can accurately assess the risk of CVD still needs more verification.

The primary aim of this study was to examine the correlation between TyG index and CVD among individuals with varying nutritional statuses, with the goal of contributing towards the prevention of CVD.

## Methods

### Study population

**Figure 1:**
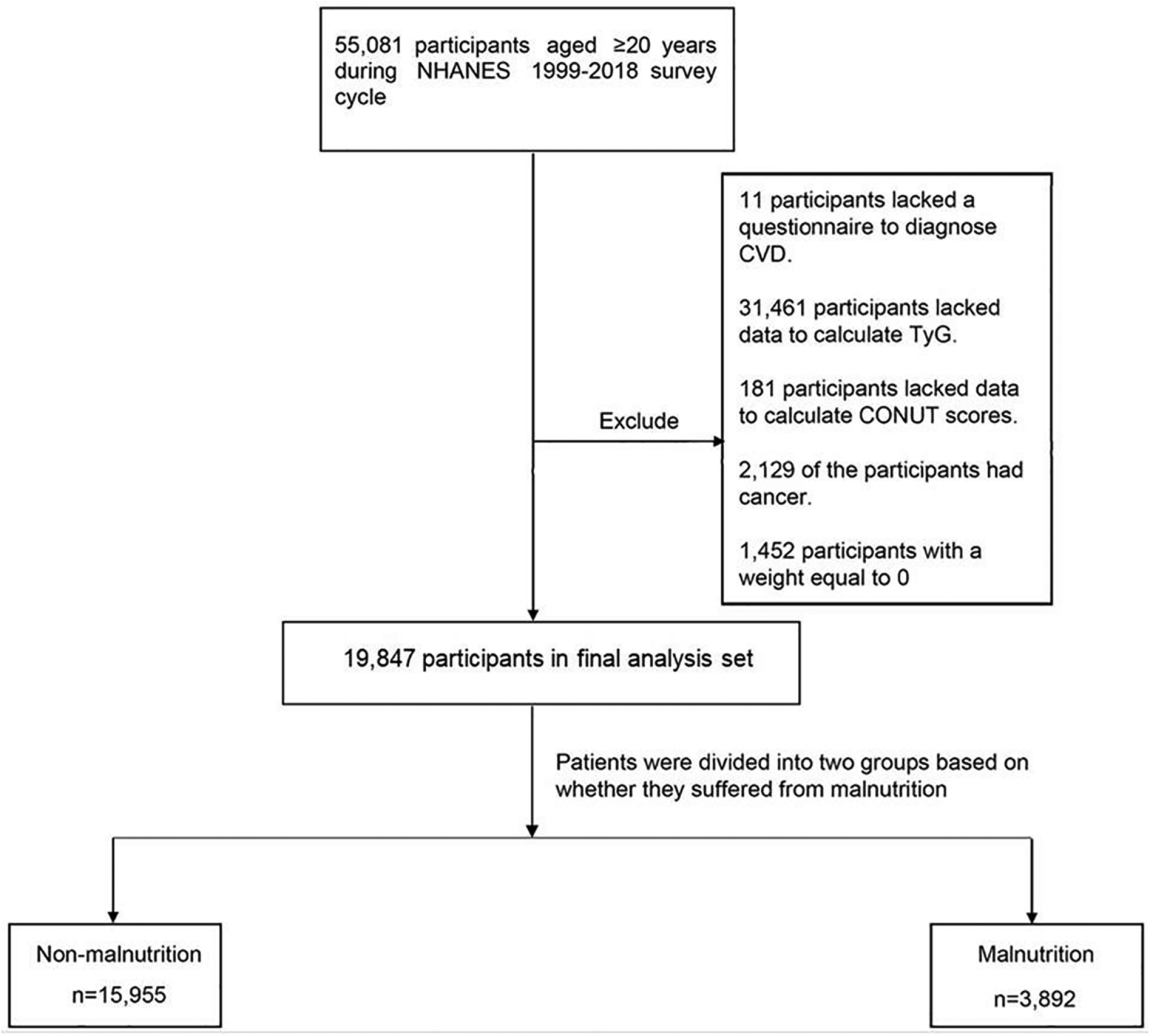
Flowchart of the study design.

This study utilized data from the National Health and Nutrition Examination Survey (NHANES). Specifically, data from the NHANES 1999–2018 cycle were examined, involving a total of 55,081 participants aged 20 years and older. Eleven participants were excluded from the study due to incomplete questionnaires for CVD diagnosis. Additionally, 31,461 participants lacked the necessary data to calculate the TyG index and were therefore excluded from the analysis. Similarly, 181 participants lacked the data required for calculating Controlling Nutritional Status (CONUT) scores and were subsequently omitted from the study. Furthermore, 2,129 participants were excluded due to pre-existing cancer conditions, and an additional 1,452 patients were removed due to having a weight of zero. Finally, the analysis encompassed a total of 19,847 participants (Figure 1).

### Calculation formula of TyG

According to the definition in other study, TyG index = ln[fasting triglyceride (TG, mg/dl) × fasting blood glucose (FBG, mg/dl)]/2 (11).

### Definition of malnutrition

The participants’ nutritional status was evaluated by implementing the CONUT score (12). The calculation method of CONUT score was shown in Supplementary Table S1. Patients with a CONUT score greater than 1 were considered to be malnourished.

### Primary outcome

The Primary outcome was CVD. Individuals who affirmed their medical history of CVD (including conditions such as coronary heart disease, congestive heart failure, heart attack, stroke, and angina) in response to the NHANES questionnaire item were classified as having CVD.

### Study groups

The quartiles of the TyG index were computed for both the undernourished and non-undernourished populations, and the patients were categorized based on these quartile calculations. For the non-undernourished population, the 25th, 50th, and 75th percentiles were 8.2, 8.6, and 9.0, respectively. In the undernourished population, the 25th, 50th, and 75th percentiles were 7.9, 8.3, and 8.7, respectively.

### Confounding variable

The participants provided self-reported information on their age, gender, race, waist-height ratio (WHtR), smoking habits, and alcohol consumption. NHANES staff collected fasting blood and urine samples to determine lipid data including FBG, Total cholesterol (TC), TG, low-density lipoprotein cholesterol (LDL-C), urinary albumin/creatinine ratio (UACR) and estimated glomerular filtration rate (eGFR). Detailed measurement techniques are available on the NHANES website. (https://wwwn.cdc.gov/Nchs/Nhanes/2001-2002/L13AM_B.htm#Description_of_Laboratory_Methodology). Homeostatic Model Assessment of Insulin Resistance (HOMA-IR) with the following formula: fasting insulin (uU/ml) × fasting plasma glucose (mmol/L)/ 22.5 (13). Participants with a WHtR ≥0.5 were considered to be affected by obesity. Individuals who met any of the following three criteria were classified as having high blood pressure: (1) Previously diagnosed by a medical professional with hypertension. (2) Systolic blood pressure ≥140 mmHg or diastolic blood pressure ≥90 mmHg. (3) Self-reported use of medication for blood pressure control. Participants were classified as having diabetes mellitus (DM) if they met any of the following conditions: (1) Hemoglobin A1c (HbA1c) >6.5%; (2) Fasting glucose levels ≥7.0 mmol/L; (3) Random blood glucose levels ≥11.1 mmol/L; (4) Blood glucose levels ≥11.1 mmol/L during a 2-hour Oral Glucose Tolerance Test (OGTT); and (5) Use of medication or insulin for diabetes management. (6) Self-reported previous diagnosis of DM by a medical professional. Individuals with a eGFR lower than 60 ml/min/1.73 m^2^ and/or a UACR higher than 30 mg/g were classified as having Chronic kidney disease (CKD). Moreover, participants who acknowledged having received a CKD diagnosis from a medical professional were also categorized as having CKD.

### Statistical analysis

Each participant in NHANES was assigned weight data, which were later used for complex sampling analysis in all statistical analyses. Means (standard error) were used to report continuous variables, while numbers (percentages) were used to report categorical variables. Baseline characteristics were compared using an analysis of variance or *t*-tests for continuous variables, and tests for categorical variables, respectively. The weights recommended by NHANES were utilized throughout the analysis.

To investigate the potential relationship between the TyG index and CVD, logistic regression analysis was employed. Model 1 was conducted without any adjustments. Model 2 included age, gender, and race as covariates. Model 3 further adjusted for age, gender, race/ethnicity, smoking habits, alcohol consumption, obesity, TC, hypertension, DM, and CKD. Restricted cubic spline (RCS) was utilized to visually illustrate the association between the TyG index and CVD. To assess whether nutritional status influenced the relationship between the TyG index and CVD, separate analyses were conducted for non-malnourished and malnourished patients. The multiplicative interaction model was employed to evaluate potential differential effects between the TyG index and nutritional status. Finally, the patients were categorized into four groups based on the median TyG index and nutritional status, and the CVD risk in these four groups was examined.

The statistical analysis was performed by the survey package in R Studio (version 4.2.1). Two-sided *p*-values <0.05 indicated significance for all analyses.

## Results

### Participant characteristics

For the analysis, a total of 19,847 individuals were considered. The average age of the participants was 45.3(0.2) years, out of which 9,589(49.03%) were male. The average TyG index of the participants was 8.6(0.0). After grouping according to nutritional status. 15,955 participants were non-malnourished and 3,892 were defined as malnourished. The TyG index [8.7(0.0).vs. 8.3(0.0)], TG [137.5(1.3) mg/dl.vs. 96.9(1.4) mg/dl], TC [202.5(0.4) mg/dl.vs. 161.0(0.9) mg/dl] and LDL-C [122.2(0.4) mg/dl.vs. 88.3(0.7) mg/dl] was observed to be notably higher among the non-malnourished group compared to the malnourished group. The prevalence of CVD [1,283(6.5%).vs. 631(13.2%)] was lower in the non-malnourished group (Table 1). Table 2 presents the baseline characteristics of participants categorized according to their TyG index quartile and nutritional status. In the non-malnourished group, as the TyG index increased, there was a higher proportion of males and smokers, and the age, FBG, TG, TC, and LDL-C levels were elevated. They were also more likely to have DM, hypertension, and CKD. Similarly, in the malnourished group, as the TyG index increased, the proportion of males, smokers, individuals with DM, hypertension, and CKD also increased. Furthermore, age, FBG, TG, TC, and LDL-C levels were higher.”

**Table 1:** Baseline characteristics of undernourished and undernourished populations (weighted).

Variable	Total (*N* = 19,847)	Non-malnutrition (*N* = 15,955)	Malnutrition (*N* = 3,892)	*P-value*
TyG	8.6 (0.0)	8.7 (0.0)	8.3 (0.0)	<0.001
HOMA-IR	3.4 (0.0)	3.4 (0.1)	3.2 (0.1)	0.115
Age, years	45.3 (0.2)	45.2 (0.2)	46.1 (0.4)	0.024
Male, *n* (%)	9,589 (49.03)	7,663 (49.0)	1,926 (49.2)	0.872
Race, *n* (%)				<0.001
Mexican American	3,789 (8.8)	3,161 (9.2)	628 (7.3)	
Non-Hispanic Black	3,949 (11.5)	3,116 (11.3)	833 (12.5)	
Non-Hispanic White	8,411 (66.5).	6,648 (66.2)	1,763 (68.2)	
Other Hispanic	1,754 (5.8)	1,464 (60.0)	290 (5.0)	
Other race	1,944 (7.3)	1,566 (7.4)	378 (7.0)	
FBG, mg/dl	104.3 (0.3)	104.3 (0.3)	103.9 (0.6)	0.533
TG, mg/dl	129.9 (1.1)	137.5 (1.26)	96.9 (1.4)	<0.001
TC, mg/dl	194.8 (0.5)	202.5 (0.4)	161.0 (0.9)	<0.001
LDL-C, mg/dl	115.8 (0.4)	122.3 (0.4)	88.3 (0.7)	<0.001
Lymphocyte, (K/μl)	2.0 (0.0)	2.1 (0.0)	1.6 (0.0)	<0.001
Albumin, g/dl	4.3 (0.0)	4.3 (0.0)	4.2 (0.0)	<0.001
Smoke, *n* (%)	8,833 (45.7)	7,163 (46.5)	1,670 (42.3)	0.002
Drink, *n* (%)	12,415 (74.7)	10,122 (75.3)	2,293 (72.4)	0.004
Affected by obesity, *n* (%)	15,788 (77.0)	12,926 (80.9)	2,862 (71.8)	<0.001
DM, *n* (%)	3,478 (13.3)	2,623 (12.3)	855 (17.4)	<0.001
Hypertension, *n* (%)	7,861 (34.7)	6,276 (34.5)	1,585 (35.4)	0.414
CKD, *n* (%)	3,225 (12.5)	2,358 (11.4)	867 (17.4)	<0.001
CVD, *n* (%)	1,914 (7.7)	1,283 (6.5)	631 (13.2)	<0.001

TyG, triglyceride-glucose index; HOMA-IR, homeostatic model assessment of insulin resistance; FBG, fasting blood glucose; TG, triglyceride; TC, total cholesterol; LDL-C, low density lipoprotein cholesterol; DM, diabetes mellitus; CKD, chronic kidney disease; CVD, cardiovascular disease.

**Table 2:** Baseline characteristics stratified according to the TyG index quartile (weighted).

Variable	Non-malnutrition					Malnutrition				
	Q1 (*N* = 3,776)	Q2 (*N* = 3,962)	Q3 (*N* = 3,990)	Q4 (*N* = 4,227)	*P*-value	Q1 (*N* = 844)	Q2 (*N* = 889)	Q3 (*N* = 1,004)	Q4 (*N* = 1,155)	*P* -value
TyG	7.9 (0.0)	8.4 (0.0)	8.8 (0.0)	9.5 (0.0)	<0.001	7.6 (0.0)	8.1 (0.0)	8.5 (0.0)	9.2 (0.0)	<0.001
Age, years	40.4 (0.4)	44.1 (0.3)	47.1 (0.3)	49.2 (0.3)	<0.001	37.9 (0.8)	43.1 (0.8)	49.4 (0.7)	54.0 (0.7)	<0.001
HOMA-IR	1.9 (0.0)	2.5 (0.0)	3.5 (0.1)	5.8 (0.1)	<0.001	1.6 (0.1)	2.4 (0.3)	3.1 (0.1)	5.8 (0.3)	<0.001
Male, *n* (%)	1,410 (38.4)	1,816 (46.1)	2,036 (52.8)	2,401 (58.6)	<0.001	351 (38.2)	421 (45.6)	561 (55.8)	593 (57.1)	<0.001
Race, *n* (%)					<0.001					<0.001
Mexican American	463 (6.6)	733 (9.2)	850 (9.6)	1,115 (11.3)		97 (6.0)	129 (7.1)	157 (7.4)	245 (8.8)	
Non-Hispanic Black	1,208 (18.8)	866 (12.3)	570 (7.6)	472 (6.4)		273 (18.3)	229 (14.2)	203 (11.2)	128 (6.5)	
Non-Hispanic White	1,392 (61.7)	1,625 (65.6)	1,768 (68.7)	1,863 (68.8)		330 (63.7)	379 (66.9)	475 (69.6)	579 (72.5)	
Other Hispanic	298 (5.6)	351 (5.9)	411 (6.6)	404 (6.1)		60 (5.7)	60 (4.4)	76 (4.9)	94 (4.9)	
Other Race	415 (7.4)	387 (7.0)	391 (7.6)	373 (7.4)		84 (6.3)	92 (7.4)	93 (6.9)	109 (7.2)	
FBG, mg/dl	93.5 (0.2)	98.0 (0.2)	102.9 (0.3)	122.9 (0.9)	<0.001	92.1 (0.4)	96.5 (0.6)	102.2 (0.7)	124.9 (1.9)	<0.001
TG, mg/dl	59.7 (0.3)	95.2 (0.3)	135.7 (0.5)	259.3 (3.1)	<0.001	44.9 (0.5)	69.5 (0.4)	97.5 (0.7)	175.5 (2.9)	<0.001
TC, mg/dl	187.1 (0.6)	198.9 (0.7)	206.2 (0.7)	217.9 (0.9)	<0.001	146.8 (1.1)	156.0 (1.7)	165.5 (1.4)	175.8 (2.0)	<0.001
LDL-C, mg/dl	111.1 (0.6)	123.5 (0.7)	128.6 (0.6)	126.2 (0.8)	<0.001	77.5 (0.9)	86.8 (1.4)	94.5 (1.2)	94.5 (1.5)	<0.001
Lymphocyte, (K/μl)	2.0 (0.0)	2.1 (0.0)	2.1 (0.0)	2.2 (0.0)	<0.001	1.6 (0.0)	1.5 (0.0)	1.5 (0.0)	1.6 (0.0)	0.018
Albumin, g/dl	4.3 (0.0)	4.3 (0.0)	4.3 (0.0)	4.3 (0.0)	<0.001	4.2 (0.0)	4.2 (0.0)	4.1 (0.0)	4.0 (0.0)	<0.001
Smoke, *n* (%)	1,368 (38.3)	1,733 (45.3)	1,843 (47.9)	2,219 (54.6)	<0.001	293 (33.1)	342 (40.0)	457 (46.5)	578 (49.5)	<0.001
Drink, *n* (%)	2,482 (78.7)	2,582 (77.1)	2,494 (74.5)	2,564 (70.9)	<0.001	555 (78.1)	553 (73.3)	577 (71.1)	608 (66.9)	<0.001
Affected by obesity, *n* (%)	2,407 (61.6)	3,129 (78.3)	3,477 (89.2)	3,913 (94.8)	<0.001	442 (51.7)	591 (64.1)	799 (79.8)	1,030 (92.1)	<0.001
DM, *n* (%)	163 (2.8)	325 (5.6)	569 (10.4)	1,566 (30.6)	<0.001	50 (4.2)	100 (8.7)	209 (15.3)	496 (43.1)	<0.001
Hypertension, *n* (%)	974 (20.6)	1,371 (29.0)	1,739 (39.3)	2,192 (49.2)	<0.001	186 (18.3)	294 (25.9)	481 (42.9)	624 (54.5)	<0.001
CKD, *n* (%)	324 (6.9)	468 (9.0)	628 (12.0)	938 (17.7)	<0.001	97 (9.7)	156 (13.5)	241 (18.9)	373 (27.5)	<0.001
CVD, *n* (%)	161 (3.2)	252 (4.7)	354 (7.1)	516 (10.9)	<0.001	59 (5.9)	111 (9.4)	190 (14.7)	271 (22.9)	<0.001

TyG, triglyceride-glucose index; HOMA-IR, homeostatic model assessment of insulin resistance; FBG, fasting blood glucose; TG, triglyceride; TC, total cholesterol; LDL-C, low density lipoprotein cholesterol; DM, diabetes mellitus; CKD, Chronic kidney disease; CVD, cardiovascular disease;.

### Relationship between TyG and CVD in patients without malnutrition

**Figure 2:**
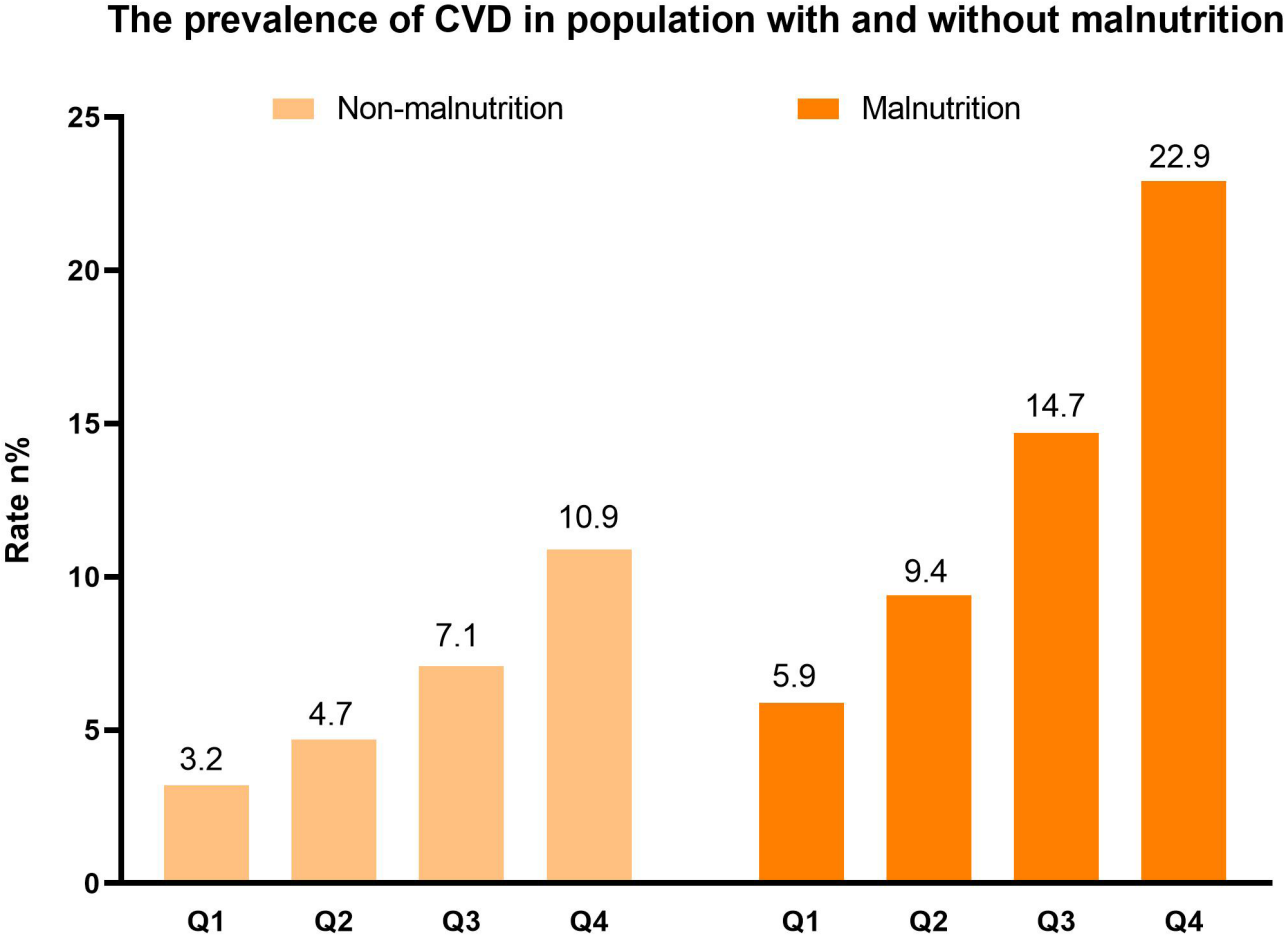
The distribution of CVD (weighted).

**Figure 3:**
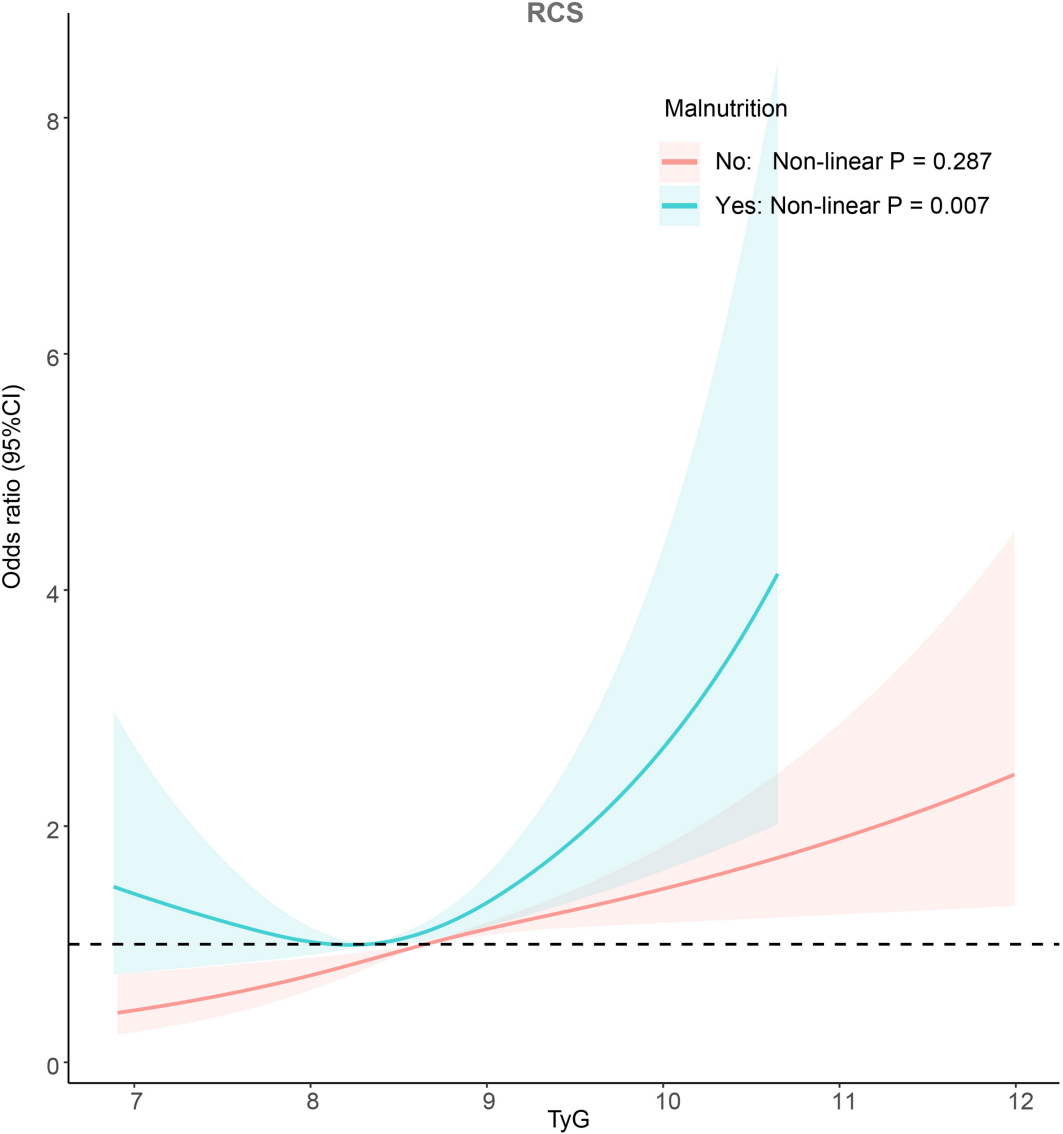
Potential non-linear relationship between TyG index and CVD (weighted).

Among individuals without malnutrition, a total of 1,283 participants were identified as having CVD. Notably, the prevalence of CVD increased in tandem with a higher TyG index (Figure 2). Univariate logistic regression analysis revealed that individuals with higher TyG index values faced an increased risk of developing CVD (Group 2: Odds Ratio (OR): 1.65; 95% confidence interval (CI): 1.04–2.61; Group 3: OR: 2.75; 95% CI: 1.80–4.19; Group 4: OR: 4.75; 95% CI: 3.23–6.96, *P* for trend <0.001). After fully adjusting for possible confounding variables, the correlation between the TyG index and CVD remained consistent. Specifically, individuals with higher TyG index level were still found to to be at a greater risk of developing CVD (Group 2: OR: 1.14; 95% CI: 0.85–1.53; Group 3: OR: 1.36; 95% CI: 1.03–1.79; Group 4: OR: 1.72; 95% CI: 1.31–2.25, *P* for trend <0.001). Detailed results were presented in Table 3. The RCS adjusted for Model 3 showed that in individuals without malnutrition, there was a positive correlation between the TyG index and CVD (Non-linear *P* = 0.287; Figure 3).

**Table 3:** The relationship between TyG index and CVD in participants with and without malnutrition.

	Non-malnutrition		Malnutrition	
	Model 1			
	OR (95% CI)	*P-value*	OR (95% CI)	*P-value*
Group 1	Reference		Reference	
Group 2	1.65 (1.04,2.61)	0.032	1.65 (1.04,2.61)	0.032
Group 3	2.75 (1.80,4.19)	<0.001	2.75 (1.80,4.19)	<0.001
Group 4	4.74 (3.23,6.96)	<0.001	4.74 (3.23,6.96)	<0.001
*P* for trend		<0.001		<0.001
	Model 2			
	OR (95% CI)	*P-value*	OR (95% CI)	*P-value*
Group 1	Reference		Reference	
Group 2	1.18 (0.89,1.55)	0.250	1.07 (0.64,1.79)	0.789
Group 3	1.59 (1.21,2.10)	0.001	1.13 (0.70,1.82)	0.617
Group 4	2.47 (1.92,3.19)	<0.001	1.67 (1.08,2.58)	0.022
*P* for trend		<0.001		0.002
	Model 3			
	OR (95% CI)	*P-value*	OR (95% CI)	*P-value*
Group 1	Reference		Reference	
Group 2	1.14 (0.85,1.53)	0.371	0.82 (0.46,1.48)	0.509
Group 3	1.36 (1.03,1.79)	0.030	0.88 (0.49,1.57)	0.661
Group 4	1.72 (1.31,2.25)	<0.001	1.45 (0.83,2.52)	0.188
*P* for trend		<0.001		0.067
*P* for interaction for TyG and malnutrition (Model 3): 0.045				

Model 1: Not adjusted.

Model 2: Adjusted by age, gender, race/ethnicity.

Model 3: Adjusted by age, gender, race/ethnicity, smoke, drink, obese, TC, Hypertension, DM and CKD.

### Relationship between TyG and CVD in patients with malnutrition

In individuals suffering from malnutrition, CVD was found in 631 cases. Similar to the non-malnourished population, those with a higher TyG index had a greater prevalence of CVD (Figure 2). According to Model 1, individuals with an elevated TyG index faced an increased likelihood of developing CVD (Group 2: OR: 1.65; 95% CI: 1.04–2.61; Group 3: OR: 2.75; 95% CI: 1.80–4.19; Group 4: OR: 4.74; 95% CI: 3.23–6.96, *P* for trend <0.001). In Model 3, only Group 4 was observed to have a higher risk of CVD, but this was not statistically significant (Group 2: OR: 0.82; 95% CI: 0.46–1.48; Group 3: OR: 0.88; 95% CI: 0.49–1.57; Group 4: OR: 1.45; 95% CI: 0.83–2.52, *P* for trend =0.067). Detailed results were shown in Table 3. The adjusted RCS of Model 3 indicated a U-shaped association between the TyG index and the risk of CVD in the malnourished population (Non-linear *P* = 0.007; Figure 3).

### Relationship between TyG and malnutrition

The results of the multiplicative interaction model show that TyG index has an interaction with malnutrition in assessing the risk of CVD In the American community population (*P for interaction** *= 0.045; Table 3).

### Combined effects of TyG index and malnutrition on CVD risk

Through univariate logistic regression analysis, it was revealed that patients with high TyG index and malnutrition had the highest risk of CVD (OR: 6.90; 95% CI: 5.59−8.51; *P* < 0.001). In Model 3, patients with a high TyG index and malnutrition remained at the highest risk of CVD (OR: 2.06; 95% CI: 1.57−2.71; *P* < 0.001, Table 4).

**Table 4:** Combined effects of TyG index and malnutrition on CVD risk (weighted).

Variable	Model 1		Model 2		Model 3	
	OR (95%CI)	*P-value*	OR (95%CI)	*P-value*	OR (95%CI)	*P-value*
Categorical variable						
Low TyG and non-malnutrition	Reference		Reference		Reference	
Low TyG and malnutrition	2.61 (2.09,3.28)	<0.001	2.28 (1.79,2.91)	<0.001	1.60 (1.20,2.14)	0.001
High TyG and non-malnutrition	2.43 (2.08,2.83)	<0.001	1.81 (1.54,2.14)	<0.001	1.51 (1.25,1.83)	<0.001
High TyG and malnutrition	6.90 (5.59,8.51)	<0.001	3.51 (2.82,4.39)	<0.001	2.06 (1.57,2.71)	<0.001

Model 1: Not adjusted.

Model 2: Adjusted by age, gender, race/ethnicity.

Model 3: Adjusted by age, gender, race/ethnicity, smoke, drink, obese, TC, Hypertension, DM and CKD.

## Discussion

This research uncovered that the relationship between the TyG index and the risk of CVD differs among individuals with different nutritional status. The RCS illustrated that in the non-malnourished group, an elevated TyG index was linked to a greater likelihood of CVD. However, in the malnourished group, a U-shaped relationship was observed between the TyG index and the risk of developing CVD. Furthermore, the multiplicative interaction model indicated an interaction between the TyG index and nutritional status, suggesting that the TyG index's ability to predict CVD was influenced by nutritional status.

Previous studies widely considered the TyG index to be a reliable indicator for assessing the degree of insulin resistance (14). As the TyG index increases, the probability of developing CVD and the risk of all-cause and CVD-related mortality also rise (15). Although the TyG index was originally developed and validated in healthy people, many studies have indicated that even in unhealthy people such as DM, hypertension and CKD, here existed a connection between the TyG index and the risk of CVD (16–18). To delve deeper into whether the relationship between the TyG index and CVD events is influenced by other health factors, many studies conducted subpopulation analyses and discussions based on age, gender and BMI (19, 20). Nonetheless, limited research has investigated whether exploring whether nutritional status affected the association between the TyG index and CVD.

Malnourished patients are more susceptible to hypoglycemia due to glycogen depletion (21). Additionally, low serum TG is also regarded as an important indicator of malnutrition (22, 23). As a result, the TyG index, derived from glucose and TG, is expected to be lower in individuals with malnutrition, in alignment with the findings of this study. It's worth noting that malnutrition can lead to insulin resistance (24), which contradicts the expectation of a lower TyG index. Therefore, further research is required to determine whether the TyG index, calculated from blood glucose and blood lipid levels, can still accurately assess the risk of CVD in different nutritional states.

The results of this study show a parallel increase in TyG index and CVD risk in non-malnourished individuals (nonlinear *P* = 0.721). Given that the TyG index was developed based on data from individuals who had normal health conditions, it is not unexpected to observe a linear relationship between the TyG index and CVD in participants without malnutrition. A substantial body of research has consistently indicated that the TyG index serves as an indicator of insulin resistance severity, and heightened levels of insulin resistance were correlated with an elevated risk of CVD (25, 26).

In malnourished individuals, although the results of logistic regression analysis indicated that the TyG index did not seem to be associated with the risk of CVD, the RCS demonstrated a U-shaped relationship between TyG and the risk of CVD (Non-linear *P* = 0.007). Consistent with the findings of this study, several studies have also observed a U-shaped relationship between the TyG index and CVD in the elderly population (19, 27). The elderly population was widely recognized as being susceptible to malnutrition, with approximately half of older individuals experiencing varying degrees of nutritional deficiency (28). Depending on the method of calculating the TyG index, a very low TyG index was likely indicative of more severe malnutrition. In recent years, several studies have demonstrated a positive correlation between the degree of malnutrition and the risk of CVD (29, 30). Furthermore, since stratification was done based on nutritional status, individuals confirmed to be nutritionally normal at the time of stratification have their nutritional indicators within the normal range. In such cases, a lower TyG index may primarily signify lower insulin resistance, rather than nutritional status. This could be the reason why U-shaped results are observed mainly in malnourished individuals.

As observed in the RCS, it appears that the malnourished population with higher TyG levels face a greater risk of CVD compared to the non-malnourished population with higher TyG levels. Therefore, the combined effects of the TyG index and nutritional status on the risk of CVD was investigated. The results indicated that individuals with an elevated TyG index and malnutrition had an increased risk of developing CVD when compared to patients with a higher TyG index and normal nutritional status. As mentioned earlier, the positive correlation between the TyG index and insulin resistance has been confirmed in healthy individuals. However, in malnourished patients, the degree of correlation between the two may be altered due to low blood lipids and low blood sugar resulting from malnutrition. As seen in the baseline data in Table 1, there was no statistical difference in HOMA-IR between participants with and without malnutrition, despite having a lower TyG index. This indirectly suggests that when the TyG index was the same, the level of insulin resistance in patients with malnutrition may be higher than that in patients with normal nutrition.

This study shows that the predictive effect of the TyG index in malnourished people is inconsistent with that in non-malnourished people. The TyG index showed a U-shaped result in predicting the risk of CVD in malnourished patients, which means that not only the value itself, but also the nutritional status of the patient should be considered when predicting the risk of CVD by the TyG index. Because nutritional status has an impact on the relationship between TyG index and the risk of CVD, clinicians should interpret TyG index results with caution, especially when dealing with malnourished patients. In malnourished patients, the TyG index may underestimate their risk of CVD because these patients may exhibit risks that are inconsistent with the index. Therefore, in actual clinical practice, doctors need to comprehensively consider the patient's TyG index and nutritional status to more accurately assess their risk of CVD. This can help physicians better guide prevention and interventions to ensure that more appropriate treatment options are provided, thereby reducing the incidence of CVD and improving patients’ quality of life. In conclusion, this study emphasize the importance of considering both the TyG index and nutritional status of patients in assessing the risk of CVD. This helps to improve personalized medicine and improve diagnostic accuracy, leading to better prevention and treatment of CVD.

## Limitations

This study has certain limitations that need to be acknowledged. Firstly, due to its cross-sectional nature, the relationship observed between the TyG index and CVD can only be regarded as an association rather than a causative one. Consequently, randomized controlled trials are necessary to validate and corroborate the findings of this study. Second, many confounding variables in the study were self-reported by patients and may have been biased from actual values. Third, in this study, FBG did not appear to be affected by nutritional status. This may be because hypoglycemia is often transient, and it is difficult to detect hypoglycemia events with only one fasting blood sample, and more long-term monitoring may be needed to determine the impact of malnutrition on FBG**.** In addition, TC was included in the CONUT score used to assess nutritional status, and TC had a strong correlation with TG, but a large number of studies have confirmed that malnutrition assessed by the CONUT score has clinical significance and can be used as a diagnostic method for malnutrition (31, 32). Therefore, this study still has certain reference value.

## Conclusions

The association between the TyG index and the risk of CVD varied depending on the nutritional states. In individuals with normal nutrition, the TyG index exhibited a positive correlation with the risk of cardiovascular disease. However, in malnourished patients, the TyG index displayed a U-shaped relationship with the risk of CVD. When using TyG index to assess the risk of CVD, stratification combined with nutritional status helps to more accurately screen patients at high risk of CVD.

## Data Availability

The raw data supporting the conclusions of this article will be made available by the authors, without undue reservation.
