# Supplementary material for: Association of triglyceride-glucose index with the prevalence of cardiovascular disease in malnourished/non-malnourished patients: a large cross-sectional study
Source: Front Cardiovasc Med. 2023 Nov 28;10:1306415. doi: 10.3389/fcvm.2023.1306415 (PMC10715243; doi:10.3389/fcvm.2023.1306415)
Supplement: Supplementary file 1 [file Table1.docx]

| **Supplementary Table 1. Controlling Nutritional Status (CONUT) score calculation.** | | | | | | | | |
| --- | --- | --- | --- | --- | --- | --- | --- | --- |
|  | |  | **Undernutrition Degree** | | | | | |
| **Parameter** | **Normal** | | | | **Light** | **Moderate** | **Severe** | |
| serum albumin(g/dl) | 3.5-4.5 | | | 3.0-3.49 | | 2.5-2.94 | | <2.5 |
| score | 0 | | | 2 | | 4 | | 6 |
| total lymphocytes(/mm^3^) | >1600 | | | 1200-1599 | | 800-1199 | | <800 |
| Score | 0 | | | 1 | | 2 | | 3 |
| Cholesterol (mg/dl) | >180 | | | 140-180 | | 100-139 | | <100 |
| Score | 0 | | | 1 | | 2 | | 3 |
| Screening total score | 0-1 | | | 2-4 | | 5-8 | | 9-12 |
